# Metronomic photodynamic therapy using an implantable LED device and orally administered 5-aminolevulinic acid

**DOI:** 10.1038/s41598-020-79067-7

**Published:** 2020-12-16

**Authors:** Izumi Kirino, Katsuhiko Fujita, Kei Sakanoue, Rin Sugita, Kento Yamagishi, Shinji Takeoka, Toshinori Fujie, Shinji Uemoto, Yuji Morimoto

**Affiliations:** 1 Department of Physiology, National Defense Medical College, Namiki 3-2, Tokorozawa, Saitama 359-8513 Japan; 2 Division of Hepatobiliary-Pancreatic Surgery and Transplantation, Department of Surgery, Graduate School of Medicine, Kyoto University, Kyoto, Japan; 3 Institute for Materials Chemistry and Engineering, Kyushu University, Fukuoka, Japan; 4 Pleiades Technologies LLC, Fukuoka, Japan; 5 Graduate School of Advanced Science and Engineering, Waseda University, Tokyo, Japan; 6 Research Organization for Nano & Life Innovation, Waseda University, Tokyo, Japan; 7 Faculty of Science and Engineering, Waseda University, Tokyo, Japan; 8 School of Life Science and Technology, Tokyo Institute of Technology, Tokyo, Japan

**Keywords:** Preclinical research, Targeted therapies

## Abstract

Metronomic photodynamic therapy (mPDT) is a form of PDT that induces cancer cell death by intermittent continuous irradiation with a relatively weak power of light for a long duration (several days). We previously developed a wirelessly powered, fully implantable LED device and reported a significant anti-tumor effect of mPDT. Considering application in clinical practice, the method used for repeated administrations of photosensitizers required for mPDT should not have a high patient burden such as the burden of transvenous administration. Therefore, in this study, we selected 5-aminolevulinic acid (ALA), which can be administered orally, as a photosensitizer, and we studied the antitumor effects of mPDT. In mice with intradermal tumors that were orally administered ALA (200 mg/kg daily for 5 days), the tumor in each mouse was simultaneously irradiated (8 h/day for 5 days) using a wirelessly powered implantable green LED device (532 nm, 0.05 mW). Tumor growth in the mPDT-treated mice was suppressed by about half compared to that in untreated mice. The results showed that mPDT using the wirelessly powered implantable LED device exerted an antitumor effect even with the use of orally administered ALA, and this treatment scheme can reduce the burden of photosensitizer administration for a patient.

## Introduction

One of the effective cancer treatments is photodynamic therapy (PDT). PDT uses a photosensitizer and light in well-oxygenated cancer tissue to generate reactive oxygen species (ROS), resulting in cancer cell death^1^. Tumor-selective treatment is possible when light can be directed only to the tumor, and PDT therefore spares patients from many of the adverse effects associated with chemotherapy, radiation, and surgery^2^. Since optical devices such as fiber optics to directly irradiate the cancer tissues are used in PDT, the clinical applications of PDT are limited to specified cancers, including skin cancers (which can be directly viewed), cancers in the upper aerodigestive tract (which can be endoscopically accessible), and brain tumors (which can be directly accessed via an operation microscope). Although PDT for tumors in deeply located organs such as the liver, pancreas, and ovaries is possible in principle, it has not been clinically applicable. This may be because the procedure involves surgical invasion, such as laparotomy, and because of concerns about complications such as organ damage and postoperative adhesion due to the high irradiation intensity (> 100 mW/cm^2^) used in conventional PDT.

A new modality of PDT for treating tumors in deeply located organs is therefore needed. One new modality that has been focused on is metronomic PDT(mPDT), in which the photosensitizer is administered repeatedly and light is irradiated during an extended period at low fluence rates^3^, resulting in apoptosis-dominant cell death^4^. The key to the therapeutic efficacy of mPDT is that the number of tumor cells dying over time must exceed the tumor's regrowth rate^5^. This implies that mPDT with a single administration of photosensitizer and low-intensity light irradiation for a long time will not induce adequate tumor cell death to achieve tumor control. Hence, repeated photosensitizer administration will be required^5^.

Conventional PDT requires a light source that is capable of delivering about 100 mW/cm^2^ of irradiation intensity, whereas mPDT only requires a low intensity of about 0.1 mW/cm^2^, thus making it possible to reduce the size of the light source. This may allow implantation of the light source inside the body and treatment of tumors in deeply located organs in body cavities (cranial, thoracic, and abdominal cavities), which has not been possible with conventional PDT. We have recently developed a micro LED light source (11 × 7 × 0.8 mm) for mPDT^6^. It is wirelessly powered with a near-field communication (NFC) system and can be completely implanted inside the body.

Since the first report on anti-tumor effects of mPDT by Wilson et al. in 2004^3^, more than 10 studies have been published^3,7–9^. A clinical benefit of mPDT has also been shown^10^. However, the previously reported anti-tumor effects in most of the animal studies are qualitative without statistical evidence. On the other hand, using the above-mentioned wirelessly powered LED device, we achieved complete eradication of more than 50% of the tumors in model mice in our previous study^6^. In that study, photofrin was used as a photosensitizer and was administered intravenously every three days. However, considering application in clinical practice, such a repeated transvenous medication protocol (regimen) would be a burden for patients.

Almost all photosensitizers, including clinically used porphyrin derivatives and phthalocyanine derivatives, are currently used by transvenous administration. The only exception is 5-aminolevulinic acid (ALA), which is a photosensitizer (or more precisely a precursor to a photosensitizer) that can be administered orally. Hence, considering the need for repeated administrations, ALA is the most desirable photosensitizer among the currently available photosensitizers.

Therefore, in this study, we verified the anti-tumor effect of mPDT using our established light system when ALA was orally administered to mice.

In the present study, a green (532 nm) LED was used since our previous study showed that the anti-tumor effect of mPDT using a green (532 nm) LED was greater than that of mPDT using a red (630 nm) LED^6^.

## Results

### Temporal changes in protoporphyrin IX (PPIX) after ALA administration

After administration to the body, ALA is transferred into cells and is metabolized into protoporphyrin IX (PPIX), which functions as a photosensitizer. Administered ALA is known to accumulate more selectively in tumor tissues than in normal tissues^11^.

To determine the amount of ALA required to exert an antitumor effect in mPDT, we investigated the relationship between ALA dose and the amount of PPIX produced. In addition, to determine the optimal irradiation period in mPDT, we investigated temporal changes in the amount of PPIX in tumors after ALA administration.

Since PPIX is also a fluorescent substance, accumulation of PPIX in the tumor can be determined indirectly by fluorescence measurement, in which the relative intensity of fluorescence of PPIX from tumors can be evaluated.

**Figure 1:**
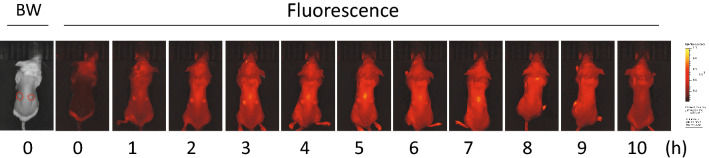
Temporal changes in PPIX fluorescence after ALA administration. A mouse with intradermal tumors (both sides of the back, indicated by dashed-line circles in the black-and-white (BW) photo) was transesophageally administered 300 mg/kg of ALA, and PPIX-derived fluorescence was measured every hour up to 10 h after administration.

**Figure 2:**
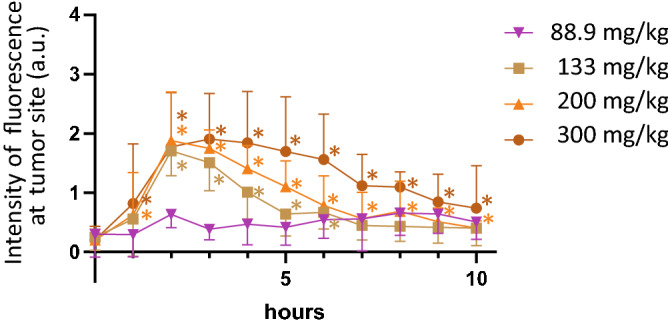
Temporal changes of relative fluorescence intensity for PPIX at tumor sites. ALA dose was 88.9, 133, 200 or 300 mg/kg per animal (n = 5 each). *; p < 0.05 compared to fluorescence values at 0 h for each curve.

An intradermal tumor model was made by implanting cancer cells at two locations in the backs of mice. ALA solution was administered transesophageally using a sonde. Figure 1 shows the temporal distribution of PPIX-derived fluorescence in the body skin of mice after administration of ALA (300 mg/kg), and Fig. 2 shows the temporal change in relative intensity of PPIX-derived fluorescence at the tumor site when the amount of ALA administered was varied.

The fluorescence intensity in systemic skin increased from 1 h after administration, and the fluorescence intensity at the tumor site was stronger and larger amounts of PPIX were produced in tumors. The fluorescence intensity at the tumor site reached the highest value at 2–3 h after ALA administration and then decreased gradually. This fluorescence trend was observed when ALA doses of 133 mg/kg or more were used, but at doses of 88.9 mg/kg, there was no significant increase in PPIX fluorescence in tumors. Peak PPIX fluorescence values were almost the same at ALA doses of 133 to 300 mg/kg, but the decline of PPIX fluorescence after the peak was more gradual with larger ALA doses.

The failure to detect PPIX fluorescence at an ALA dose of 88.9 mg/kg may be due to the experimental method in which tumor-derived fluorescence is captured through the skin. Fluorescence of PPIX produced in skin tissues became background noise, probably having masked the weak fluorescence from PPIX produced inside tumors.

Based on the above-described results, the ALA dose was set to 200 mg/kg in subsequent mPDT experiments. It was reported that toxicity to an organism is less likely to occur at lower ALA doses^3^, but the antitumor effect of mPDT was anticipated not to be fully derived when applied the minimal ALA doses with evidence of elevated PPX fluorescence (133 mg/kg).

When ALA was administered at a dose of 200 mg/kg, the fluorescence intensity in the tumor site up to 10 h after administration was significantly higher than that at 0 h. In other words, PPIX accumulated at the tumor site until at least 1–10 h after ALA administration. However, the fluorescence intensity at 7 h after administration was almost a plateau. Accordingly, in the mPDT experiment, the LED device was set to emit light until 8 h after ALA administration.

### Antitumor effects of metronomic PDT using a wirelessly powered implantable LED device and ALA administration

#### Stability of the luminescence of wirelessly powered implantable LED devices inside animals

First, to confirm the stability of luminescence of the wirelessly powered implantable LED device in a mouse, the device was implanted into the subcutaneous cavity just below the tumor to monitor the light emission.

**Figure 3:**
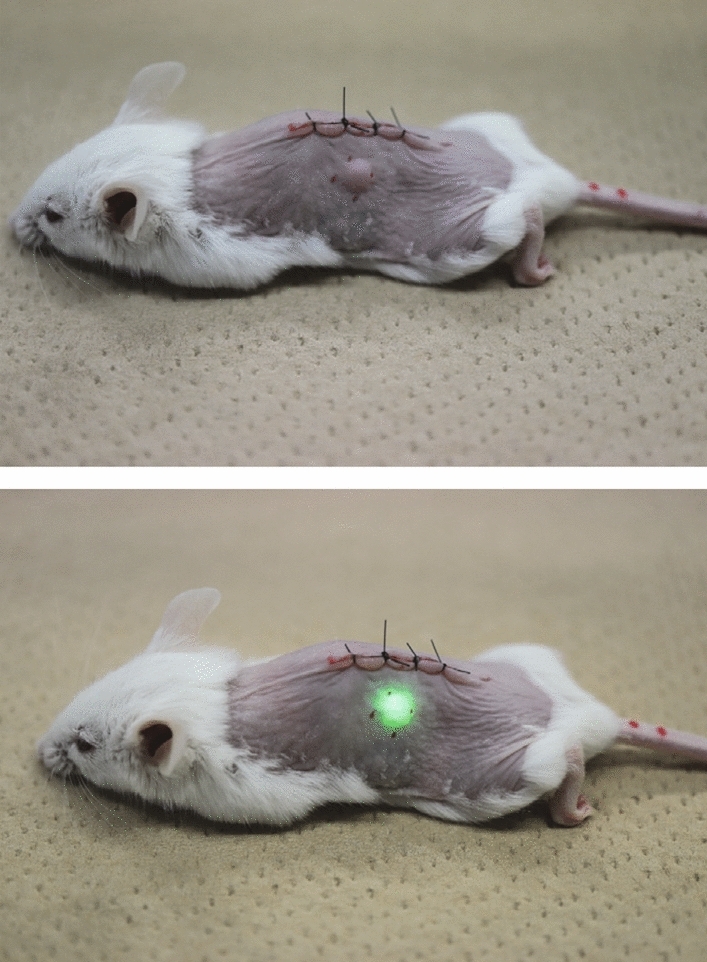
A mouse implanted with a wirelessly powered LED device in subcutaneous tissue just below the intradermal tumor: non-luminescent state (upper) and emitting state (lower).

When a mouse in which the LED device had been implanted was placed on an antenna board and the wireless power supply was started, the LED emitted light (Fig. 3). Next, the mice with implanted LED devices were transferred to a breeding cage where they could move around freely and the cage was placed on the antenna board to continue wireless power feeding. Even in this condition, the intensity of luminescence was about the same: the LED emitted light for more than 90% of the time regardless of the position of the mouse in the cage (Supplementary information video). The reason why the LEDs did not emit light for about 10% of the observation time was assumed to be reduction in transmission efficiency resulting from the relative position between the LED device and the antenna board.

#### Antitumor effect of mPDT

In order to evaluate the anti-tumor effects of mPDT using the wirelessly powered implantable LED device and ALA administration, animals were divided into the following four groups.

➀ Untreated (no ALA administration, no light exposure): ALA(−)photo(−) group.

➁ ALA administration only (no light exposure): ALA( +)photo(−) group.

➂ Light exposure only (no ALA administration): ALA(−)photo( +) group.

➃ mPDT (ALA administration and light exposure): ALA( +)photo( +) group.

**Figure 4:**
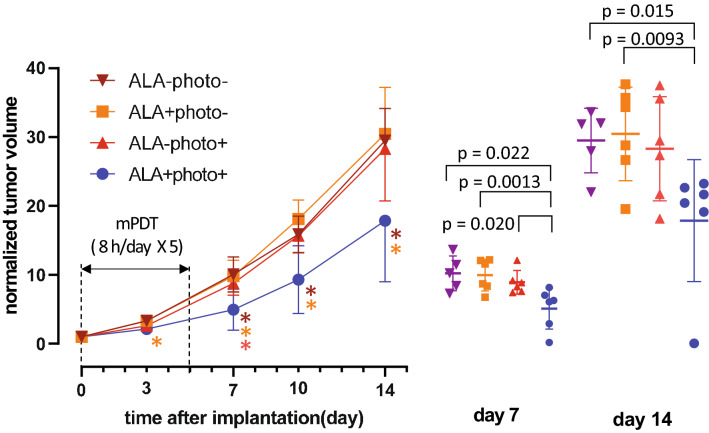
Tumor growth curve. Normalized tumor volume indicates the relative volume when the tumor volume at day 0 (at the start of mPDT) is set to 1. The mPDT was performed during the period shown in the graph. *; p < 0.05 compared to groups of animals shown in each color. The right side shows a volumetric scatter plot of individual tumors on day 7 and day 14.

Figure 4 shows the change in each tumor size from the start of treatment to day 14 in the four groups of mice. On day 7, two days after the end of mPDT, the tumor size in animals in the mPDT group ➃ was significantly smaller than the tumor sites in animals in the other groups (➀➁➂), and on day 14 (9 days after the end of treatment), the tumor size in the mPDT group was about half of the tumor sizes in the other groups. Moreover, one out of 6 animals in the mPDT group showed tumor disappearance.

**Figure 5:**
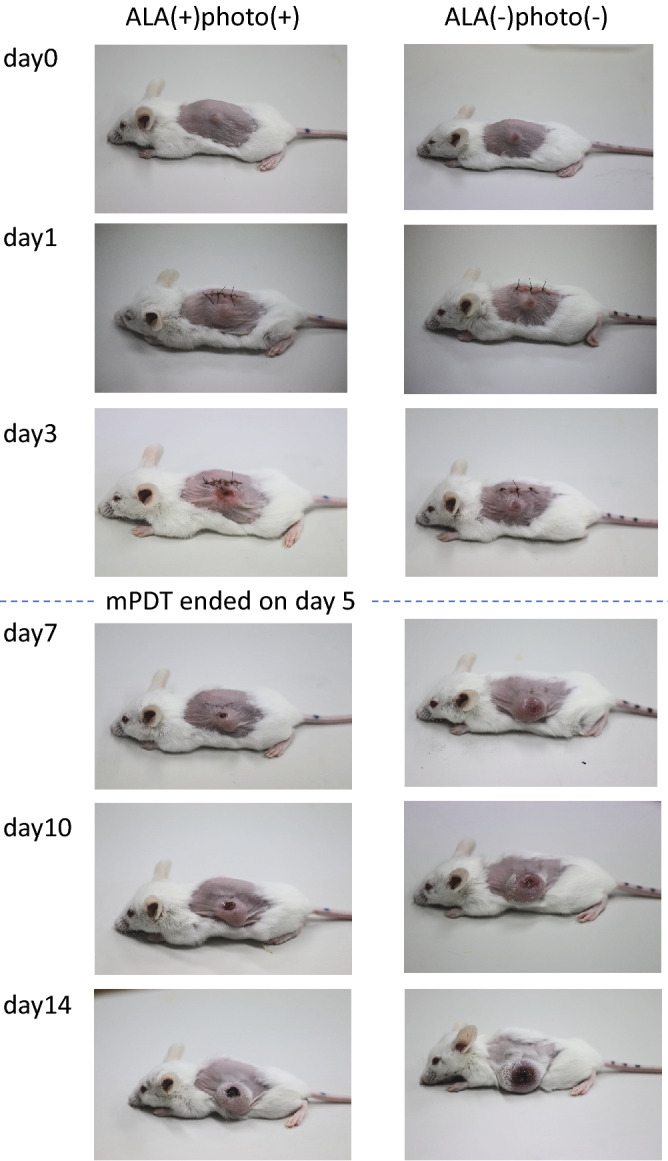
Representative photographs showing temporal changes in tumor morphology.

Figure 5 shows representative aspects of tumors in the animals in groups ➃ and ➀. In a mPDT-treated animal in group ➃, the tumor mass seemed to have almost disappeared on day 3. Thereafter, the residual tumor in the peripheral area distant from the center of light irradiation began to grow, and the tumor gradually increased donut-shapely.

#### Histopathology of mPDT-treated tumors

**Figure 6:**
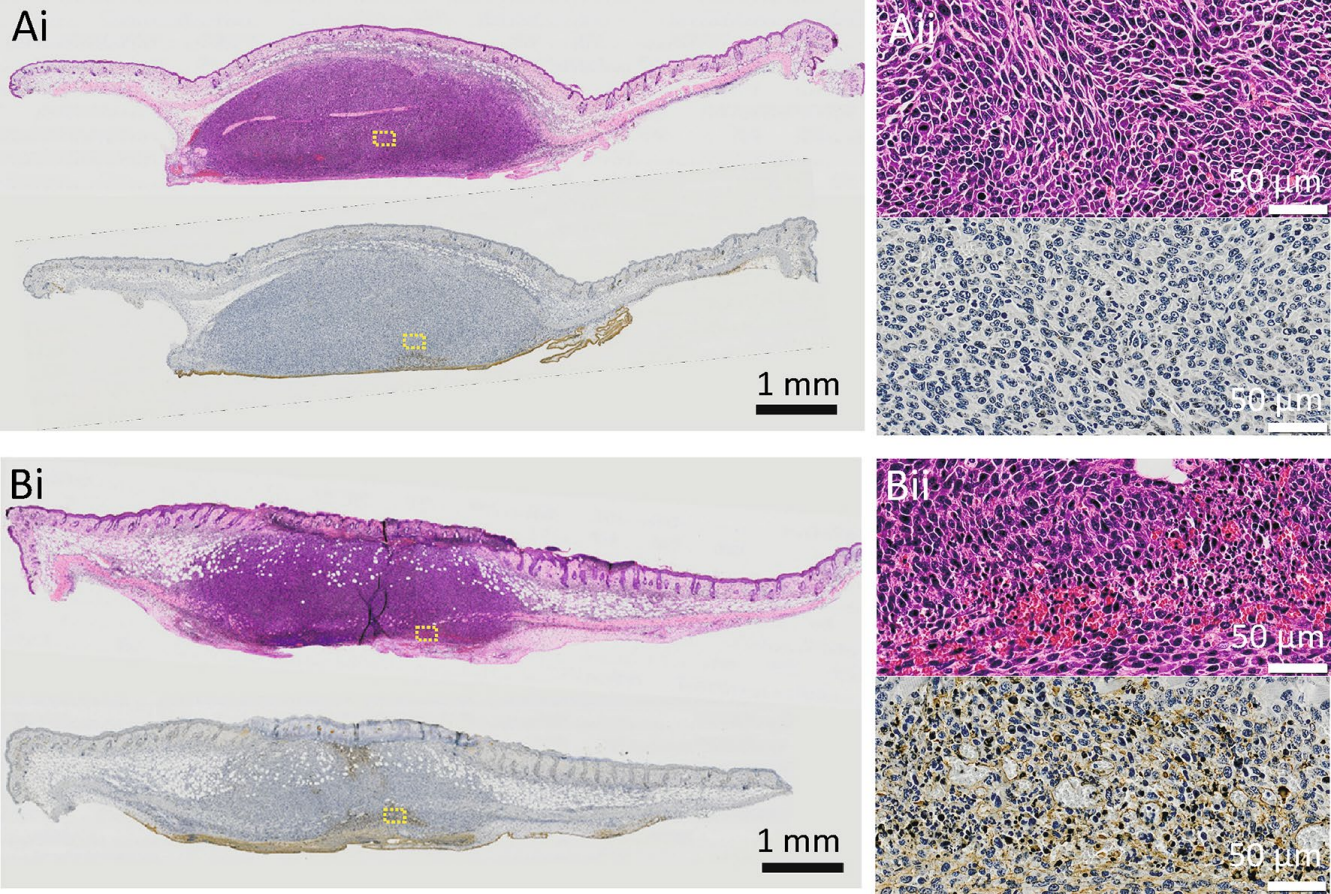
Histopathological photographs (H&E staining (upper) and Tunel staining (lower)). **(Ai)** Day 3 of an untreated tumor (ALA(−)photo(−)). A non-emitting implantable LED device had been placed at the bottom of the photograph. **(Bi)** Day 3 of an mPDT-treated tumor (ALA( +)photo( +)). An implantable LED device had been placed at the bottom of the photograph, and LED light had been illuminated approximately in the center of the tumor. Photographs of **(Aii,Bii)** are enlarged views in areas indicated as yellow-line squares in photographs of **(Ai,Bi)**.

Figure 6 shows histopathological (H&E and Tunel staining) images of a tumor on day 3 after the start of mPDT and an untreated tumor. The mPDT-treated tumor showed cell death (Tunel-positive) in its central region, and cancer cell proliferation in the central part of the tumor was inhibited compared to that in the untreated tumor. An enlarged view of the area with cell death showed nuclear condensation and cellular condensation (HE), suggesting the occurrence of apoptosis.

### Measurement of heat production by the wirelessly powered implantable LED device

**Figure 7:**
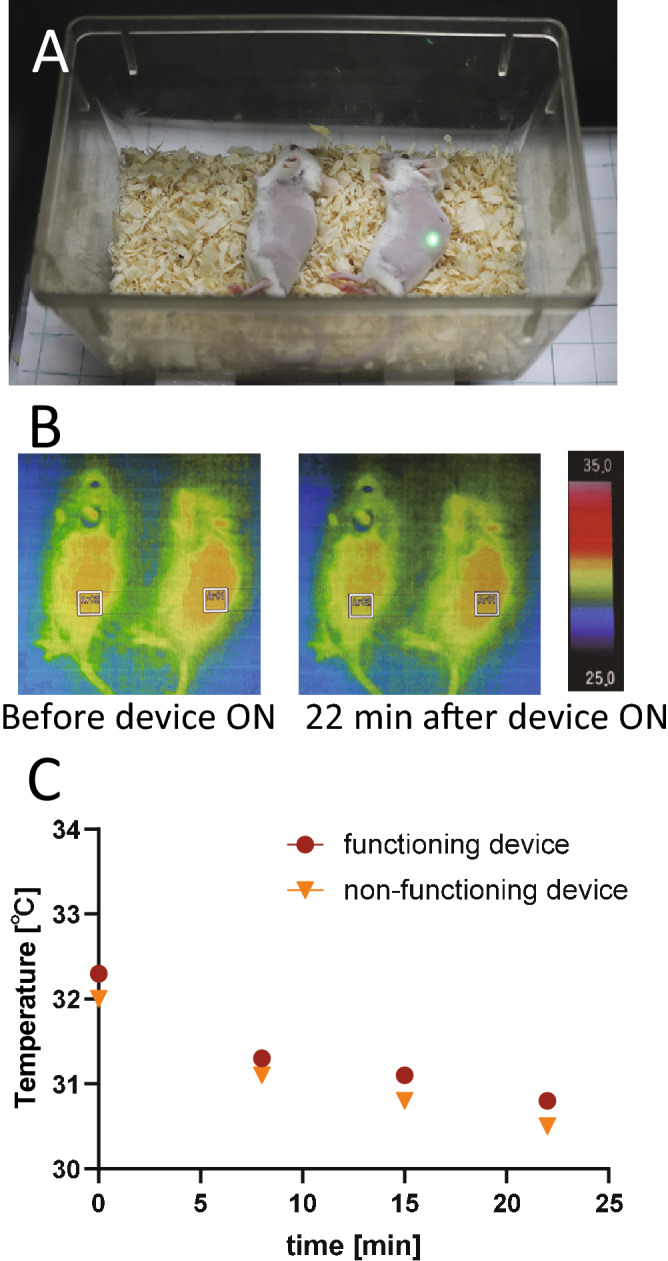
Confirmation that no heat was generated by the wirelessly powered implantable LED device. **(A)** Mice under anesthesia in a cage placed on the antenna board. The mouse on the right was implanted with a functioning device, while the mouse on the left was implanted with a nonfunctioning device in which a current did not flow through the receiver coil for the radio feeding. **(B)** Images of thermography. Square frames (white color line) indicate the location of the implanted device. **(C)** Temperature change in the skin covering the device.

A wirelessly powered LED device that emitted light was implanted into a subcutaneous cavity in a mouse and a broken device that did not emit light was implanted in another mouse. Both mice were placed on the antenna board under anesthesia, and the skin temperature was measured for 22 min using thermography after the wireless power supply was started (Fig. 7). The results showed that there was no significant difference between skin temperatures of the two mice. Apart from this experiment, the temperature of the emitting LED in the device under an unimplanted condition was measured, and tangible heat generation was not seen (Fig. S1). These results suggested that heat production by the LED device had no effect on tumor growth.

## Discussion

In the present study, mPDT with transoral administration of ALA (200 mg/kg) using a wirelessly powered fully implantable LED device with a power of about 0.05 mW induced a significant antitumor effect in intradermal tumor model mice. There was no temperature increase due to operation of the device, suggesting that the antitumor effect was due to the photochemical effects, not hyperthermia.

We believe that the ALA dosage and the wavelength of light are important factors that allowed us to show a robust antitumor effect of ALA-mPDT.

A possible reason for the lack of significant differences in the antitumor effect in previous studies is that the ALA dosage was low. ALA doses in most studies were about 100 mg/kg^3,8,9^. Based on the results of the present PPIX measurements (Fig. 2), the amount of PPIX in the tumor after administration of 100 mg/kg ALA may not be sufficient to induce a significant anti-tumor effect due to the photochemical reaction. Furthermore, at a dose of 100 mg/kg, the duration of PPIX existence in the tumor may be short (2–3 h). In the present study, by setting the ALA dose to 200 mg/kg, both the amount of PPIX and duration of PPIX existence in the tumor were considered to be sufficient for an anti-tumor effect to be exerted.

On the other hand, based on the results of PPIX measurement in this study (Fig. 2), it can be predicted that the anti-tumor effect of mPDT would be increased by further increasing the ALA dosage. However, when considering clinical application, harmful effects caused by increased ALA dosage may be a problem. In the present study, apparent changes in appearance and behavior were not observed in mice that were administered 200 mg/kg/day 5-ALA for 5 days, but a weight loss after 10 days of treatment with 300 mg/kg/day or more of ALA was reported^3^. Hence, we will explore the mPDT-relevant conditions (e.g., number of ALA doses, fluence rate of light irradiation) to produce a significant antitumor effect even when ALA is administered at a dose of less than 200 mg/kg.

With respect to the wavelength of the light source, most previous mPDT studies using porphyrin derivatives (containing PPIX) as photosensitizers used a wavelength of 630–635 nm^3,7–10,12^, which is similar to that used in conventional PDT. However, we used a green (532 nm) light in the present study. This is because our previous study showed that the anti-tumor effect of mPDT using a green (532 nm) LED was greater than that of mPDT using a red (630 nm) LED^6^. It has also been shown in a clinical study on conventional PDT that the anti-tumor effect by using a green (514 nm) light was comparable to that by using a red (630 nm) light^13^.

With respect to the tissue penetration depth of light, green (532 nm) light is more unfavorable than red (630 nm) light because the tissue penetration depths are 0.68 mm and 1.05 mm, respectively^14^. On the other hand, the absorbance of PPIX at 532 nm is 2.28 times greater than that at 630 nm. Using only these two parameters (penetration and absorption), from a simple simulation of light attenuation when light of each wavelength enters the tissue where PPIX is present, it is estimated that the light intensity is the same at a depth of about 1.5 mm.

Moreover, the extended duration of light exposure may be a reason for the superior anti-tumor effects seen in green light in the case of mPDT. Hartl et al. showed that LD_100_ fluence (a fluence of 100% lethal to cancer cells) in ALA-mPDT in the case of irradiation duration for 12 h required 10 J/cm^2^ when the light wavelength was 632 nm, while only 1/10 of LD_100_ fluence (1 J/cm^2^) was required when the light wavelength was 405 nm^15^. Hence, when 532 nm light is used, the LD_100_ fluence is estimated to be about half of that at 632 nm (about 5 J/cm^2^). This suggests that the same anti-tumor effect is obtained with green light at half the fluence rate (W/cm^2^) of that of red light in mPDT under the condition of prolonged light irradiation.

Considering the above discussion, in mPDT using PPIX as a photosensitizer, 532 nm light may be more effective than 630 nm light when treating tumors with a thickness of about 1.5 mm.

In this study, there are two limitations: one is the tumor size and the other is dosage of ALA.

If the tumor size at the start of mPDT is larger than the range set in this study (35–55 mm^3^), a sufficient anti-tumor effect may not be achieved.

This is because the LED device used in this study was a point light source, and the light-emitting area is only about 1 mm^2^ (rectangle shape with each side being about 1 mm). The light therefore does not sufficiently reach inside the tumor when the tumor is large. This problem, however, might be solved by using a planar lighting device^12^ or an illuminating device of which the surface is covered with many point-LEDs.

The dosage of ALA needs to be reconsidered for clinical use.

The 200 mg/kg dose of ALA used in this experiment is considered to be too much as a human dose since it was reported that some complications such as vomiting, skin photosensitivity, and postural dizziness (hypotension) occurred when using a dose of 60 mg/kg^16^. The ALA dosage used in conventional PDT in humans is 20 mg/kg^17^ and tumor regression at that dose has been confirmed^18^. Hence, an ALA dose of 20 mg/kg might work well in mPDT in humans. Furthermore, since drug metabolism in humans is slower than that in mice^19^, the strategy for mPDT may be more advantageous than that for conventional PDT since a prolonged duration of PPIX existing in the human body after ALA administration is expected.

Furthermore, concomitant use of drugs that inhibit PPIX metabolism and/or transport such as iron chelating agents^20^ and ABCG2 inhibitor^21^ may be effective for increasing PPIX concentration.

In summary, we orally administered ALA to mice with intradermal tumors and measured PPIX-derived fluorescence at the tumor site. When an ALA dose of more than 200 mg/kg was used, a significant increase in fluorescence from 1 to 10 h after ALA administration was confirmed. Metronomic PDT (8 h/day for 5 days) using a fully implantable wirelessly powered LED device (532 nm, 0.05 mW) at an ALA dose of 200 mg/kg suppressed tumor growth by about half compared to that in the untreated group. This study demonstrated an antitumor effect of mPDT even with orally administered ALA, bringing us one step closer to practical application of mPDT with less patient burden.

## Materials and methods

### Wirelessly powered implantable LED device and power supply system

As a wirelessly powered implantable LED device, NFC (near-field communication) LED chips (Kyoritsu Electronics Industry, Osaka, Japan; model: KP- NFLEG (λ = 530 nm), size: 7.0 × 11.0 × 0.8 mm, weight: ~ 20 mg) were encapsulated with a UV-curable resin based on urethane polymer (STAR Lab Cosmetics, Osaka, Japan).

A handmade antenna board was used for the wireless power supply. An electromagnetic induction system was used for the power supply system with a resonance frequency of 13.56 MHz and the transmission power was 3 W. The antenna board can provide power supply with a transmission distance ranging from 0 to 105 mm from the upper side of the antenna board. The size of the antenna board was 310 mm (w) × 230 mm (d) × 105 mm (h), which was sufficient for covering the size of a mouse cage.

The central wavelength of light emitted from the LED device was confirmed using a photonic multichannel spectral analyzer system (PMA-11, Hamamatsu Photonics K.K., Hamamatsu, Japan). The light intensity of each LED device was measured using a power meter with a photo-diode sensor (PD300-UV, Ophir, Saitama, Japan) and LED devices with a power of 50–60 µW were selected and used for the mPDT experiment. Devices in which wires had been mechanically broken were used as non-emitting devices for controls.

### Tumor model animals and ALA administration

#### Cell line used and culture method

A murine Colon26 (C26) cell line (kindly supplied by the National Cancer Center (Tokyo, Japan)) was used in the subsequent experiments. The cells were cultured in DMEM medium (Sigma-Aldrich, St. Louis, MO) supplemented with 10% heat-inactivated fetal bovine serum (Life Technologies, Carlsbad, CA), 100 U/mL of penicillin, 100 µg/mL of streptomycin and 0.25 µg/mL of amphotericin B (Antibiotic–Antimycotic, Life Technologies) at 37 °C in 5% CO_2_ with 95% humidity.

#### Mouse intradermal tumor model

Female Balb/c mice at 7–8 weeks of age (~ 20 g) (Japan SLC, Hamamatsu, Japan) were fed under specific pathogen-free conditions. All of the experimental protocols were approved by the National Defense Medical College Animal Care and Use Committee. All procedures were performed in accordance with the guidelines for proper conduct of animal experiments stated by the Science Council of Japan and the protocols were approved by the National Defense Medical College Animal Care and Use Committee.

To establish an experimental intradermal tumor model, mice were injected with 5 × 10^5^ C26 cells suspended in 50 µL of phosphate-buffered saline into the skin of the back under anesthesia with a mixture of midazolam (0.3 mg/kg), medetomidine (4 mg/kg) and butorphanol (5 mg/kg)^22^ that was intraperitoneally administered.

For the experiment to measure the amount of PPIX (described below), two tumors were grown in order to improve visibility in fluorescence imaging. On the other hand, for the mPDT experiment (described below), only one tumor was grown.

When the tumor of the back was evident because of cancer cell growth (size of each tumor being approximately 35–55 mm^3^), the mice underwent the following procedures.

#### Method of ALA administration

A sonde was inserted into the mouth of each mouse and a solution of 5-aminolevulinic acid (hydrochloride) (ALA) (Namiki Shoji Co., Tokyo, Japan) dissolved in distilled water (15 mL/kg, about 300 µL/animal) was transesophageally administered via the sonde. The concentrations of ALA used were at 6.0, 9.0, 13.3, and 20.0 mg/mL, corresponding to 88.9, 133, 200, and 300 mg/kg (weight of ALA per kg of the mouse body).

### Measurement of the amounts of PPIX in the tumors of mice

Since PPIX is also a fluorescent substance, accumulation of PPIX in the tumor can be determined indirectly by fluorescence measurement. Fluorescence from PPIX was visualized using an in vivo imaging system (IVIS Lumina series III, PerkinElmer) with the filter set of Ex.530 nm / Em. 630 nm under temporal anesthesia with isoflurane inhalation. The amount of fluorescence from tumors was calculated using Living Image Software 3.0 (PerkinElmer).

### Implantation of the implantable LED device and treatment protocols for mPDT

Each mouse bearing a tumor (35–55 mm^3^) was anesthetized by intraperitoneal injection of a mixture of midazolam, medetomidine and butorphanol. A midline incision (2 cm long) was made in the dorsal skin. The fascia beneath the tumor and subcutaneous tissue including the tumor were detached from each other so that the area size of the resultant lacuna was the same as that of the implantable LED device. The device was then implanted into the lacuna cavity so that the center of the LED was placed just beneath the center of the tumor. The procedure of area-size matching between the device and the lacuna was important to allow the LED not to deviate from the tumor position during mPDT. Finally, the dorsal skin was closed.

The animals were divided into the following four groups for the experiment.

➀ Untreated (no ALA administration, no light exposure): ALA(−)photo(−) group (n = 5).

➁ ALA administration only (no light exposure): ALA( +)photo(−) group (n = 6).

➂ Light exposure only (no ALA administration): ALA(−)photo( +) group (n = 6).

➃ mPDT (ALA administration and light exposure): ALA( +)photo( +) group (n = 6).

On day 0, we set up a mouse cage on top of an antenna board, and in groups ➂ and ➃, the LED device was made to emit light at approximately 50 µW. In the experimental setup, the only structure separating the LED from the tumor tissue was a dermal tissue-derived thin layer with a thickness of approximately 100 µm. Hence, the light attenuation by the thin layer can be calculated to be about 15% based on a previous report^14^. As a result, the light power at the tumor surface is estimated to be 43 µW.

The duration of luminescence was set to 8 h per day. For group ➃, the LED device was set to emit light after the administration of ALA solution. The mice in groups ➁ and ➃ were administered ALA solution (200 mg/kg) at 9 AM on day 0 and all following days (at 9 AM) until day 4 (administration for a total of 5 times). Non-emitting devices were implanted into mice in groups ➀ and ➁.

A preliminary study showed that in the mice showing uncomplete eradication of tumors even after mPDT for 8 h/day over a period of 5 days, residual tumors in the surrounding margin away from the position of LED began to regrow. This finding suggested that the light emitted from the LED no longer reached the limbic regrowing tumor, and we therefore decided on a protocol of 8 h/day for 5 days.

### Measurement of tumor size and histopathological examination of tumors

Tumor size was measured by placing a caliper on the skin and measuring the longest diameter (*l*), shortest diameter (*s*) and height (*h*). The tumor volume was defined as* l* x *s* x *h*. Measurements were performed on days 0, 3, 7, 10 and 14.

### Measurement of heat production from the implantable devices

The inevitable heat production associated with emission of light from the wirelessly powered implantable LED device when the device was placed inside the subcutaneous tissue was estimated by measuring the skin temperature at the time of LED emission. Infrared thermography (FSV-2000, Apiste, Osaka) was used for temperature monitoring. Apart from this measurement, the temperature of the emitting LED in the device under an unimplanted condition was also measured.

### Statistical analysis

Data are presented as means ± standard deviation. Statistical analysis using JMP Pro ver 14 was performed by one-way analysis of variance, and group comparisons were performed using Tukey–Kramer's HSD test. A p value of less than 0.05 was considered statistically significant.

## Supplementary Information

Supplementary Video 1.

Supplementary Figure S1.
