# Supplementary material for: Metronomic photodynamic therapy using an implantable LED device and orally administered 5-aminolevulinic acid
Source: Sci Rep. 2020 Dec 16;10:22017. doi: 10.1038/s41598-020-79067-7 (PMC7744509; doi:10.1038/s41598-020-79067-7)
Supplement: Supplementary file 2 — Supplementary Figure S1. [file 41598_2020_79067_MOESM2_ESM.pdf]

Supplementary Information

Title

Metronomic photodynamic therapy using an implantable LED device and orally administered 5-aminolevulinic acid

Authors

Izumi Kirino[1,2], Katsuhiko Fujita[3], Kei Sakanoue[4], Rin Sugita[5], Kento Yamagishi[6], Shinji Takeoka[7], Toshinori Fujie[6,8], Shinji Uemoto[2], Yuji Morimoto[1*]

[1]Department of Physiology, National Defense Medical College, Japan

[2]Division of Hepatobiliary-Pancreatic Surgery and Transplantation, Department of Surgery, Graduate School of Medicine, Kyoto University, Japan

[3]Institute for Materials Chemistry and Engineering, Kyushu University, Japan

[4]Pleiades Technologies LLC, Japan

[5]Graduate School of Advanced Science and Engineering, Waseda University, Japan

[6]Research Organization for Nano & Life Innovation, Waseda University, Japan

[7]Faculty of Science and Engineering, Waseda University, Japan

[8]School of Life Science and Technology, Tokyo Institute of Technology, Japan

Correspondence

Yuji Morimoto,
Department of Physiology, National Defense Medical College,
Namiki 3-2, Tokorozawa, Saitama 359-8513, JAPAN
Tel. +81-429-95-1483
Email: gazy246@gmail.com

(Supplmentary_video.wmv)

Supplementary video. A mouse implanted with the wirelessly powered LED device moving freely in a cage on the antenna board (invisible).

(Fig. S1)

Heat generated from the functioning LED device and the non-functioning (broken) LED device was measured by thermography while wireless power was supplied from the antenna board. There was no significant difference between the temperature of the LED in the functioning LED device and the temperature of the LED in the broken LED device.

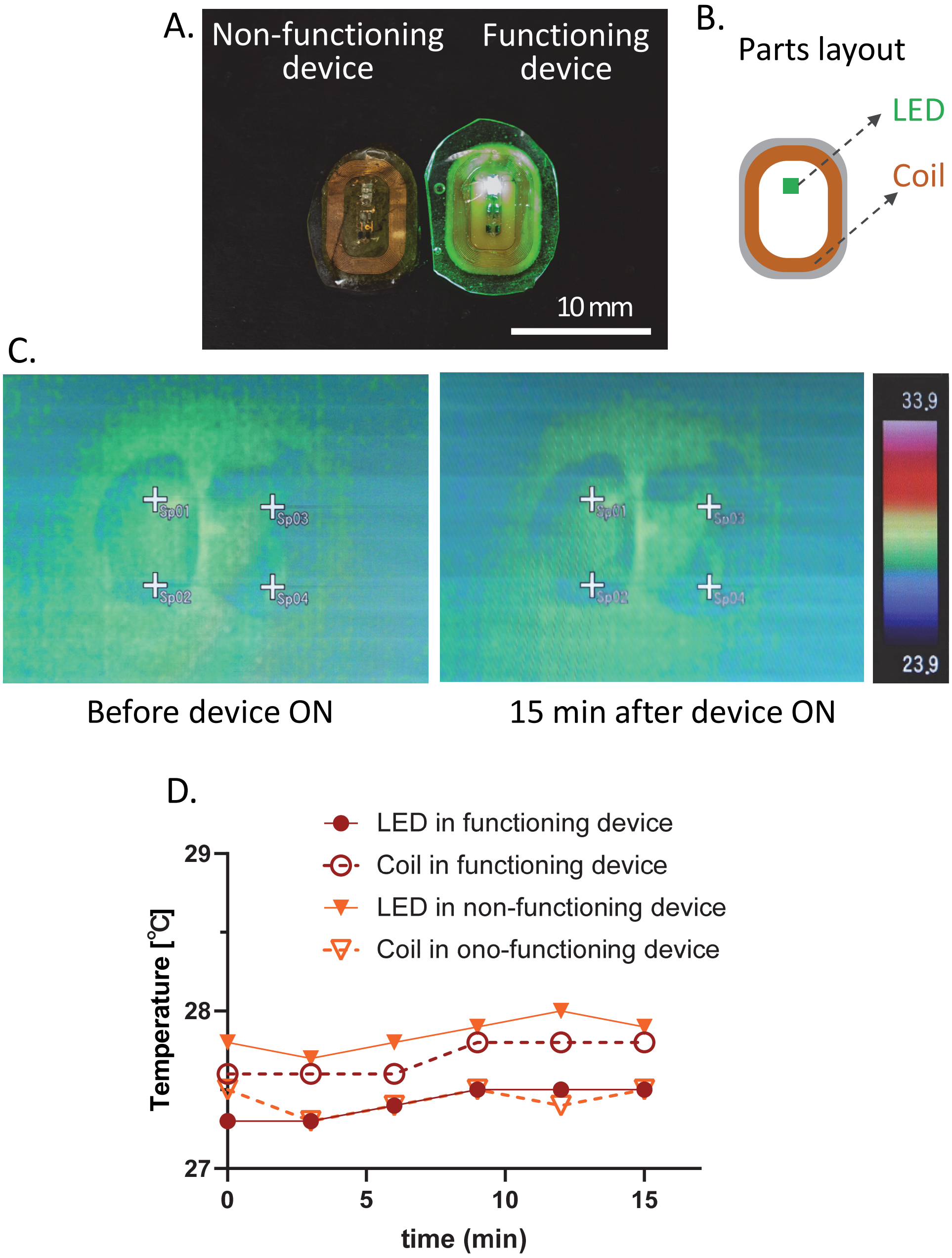

Fig. S1. Confirmation that no heat was generated from the wirelessly powered implantable LED device.
(A) Resin-encapsulated LED devices placed on the antenna board. The right one was a functioning (light illuminating) device and the left one was a non-functioning (no light illuminating) device. (B) Layouts of the LED and the coil for receiving electric power. (C) Images of thermography. Upper crosses indicate the location of the LED and lower crosses indicate the location of the coil. (D) Temperature changes in each location of the LED devices.
